# Early-Life Exposure to Lead Induces Cognitive Impairment in Elder Mice Targeting SIRT1 Phosphorylation and Oxidative Alterations

**DOI:** 10.3389/fphys.2017.00446

**Published:** 2017-06-29

**Authors:** Lijie Zhang, Runqi Tu, Yawei Wang, Yazhen Hu, Xing Li, Xuemin Cheng, Yanyan Yin, Wenjie Li, Hui Huang

**Affiliations:** College of Public Health, Zhengzhou UniversityZhengzhou, China

**Keywords:** lead (Pb), cognition, sirtuin 1(SIRT1), oxidative stress, β-amyloid (Aβ), brain derived neuronal factor (BDNF)

## Abstract

Pb is a potential risk factor for cognition, mainly mediated by enhanced oxidative stress. Resveratrol, a natural polyphenol with crucial anti-oxidative property, is recently implicated in preventing cognitive deficits in normal aging and neurodegenerative disorders. Its beneficial effects have been linked to sirtuin 1(SIRT1) activation. The aim of this work is to investigate the possible linkage between alterations in Pb-induced oxidative damage and cognitive impairment by prolonged treatment of resveratrol. Male C57BL/6 mice were given Pb(Ac)_2_ treatment or deionized H_2_O for 12 weeks, and subjected to resveratrol gavage at the dose of 50 mg/kgBw•d or vehicle after Pb exposure. Results from biochemical analysis and immunohistofluorescence showed that Pb induced oxidative DNA damage and decreased cortical antioxidant biomarker. As expected, these abnormalities were improved by resveratrol treatment. Morris water maze test, Western blotting, immunohistofluorescence staining and RT-qPCR indicated that resveratrol ameliorated spatial learning and memory deficits with alterations in hippocampal BDNF-TrkB signaling, promoted nuclear localization and phosphorylation of hippocampal SIRT1, partly increased protein levels of AMPK and PGC-1α involving in modulation of antioxidant response in Pb-exposed mice. Our results support the hypothesis that resveratrol could attenuate Pb-induced cognitive impairment which was associated with activating SIRT1 via modulation of oxidative stress. Additionally, resveratrol also repressed the Pb-induce amyloidogenic processing with resultant decline in cortical Aβ_1−−40_. Noteworthy, such effects were not mediated by resveratrol treatment alone. These findings emphasize the potential of SIRT1 activator as an efficacious dietary intervention to downgrade the Pb-induced neurotoxic lesion.

## Introduction

Heavy metal lead (Pb) is a ubiquitous environmental pollutant and has been reported to induce widespread neurotoxicity (Baranowska-Bosiacka et al., 2012; Karri et al., 2016). There is currently no doubt about the serious effect of Pb-induced oxidative stress posing to the central nervous system (CNS). Pb exposure in rodents is linked to persistent alterations of brain derived neuronal factor (BDNF), β-amyloid (Aβ) aggregation, and oxidative damage accompanied by elevated apoptotic markers (Gu et al., 2011; Hossain et al., 2016). Childhood Pb poisoning continues to pose environmental public health challenges due to tightly association with impaired intelligence and growth (Wang et al., 2012; Mostafa et al., 2016). Our previous study also showed developmental exposure to Pb caused excessive Pb accumulation in the hippocampus and dose-related cognitive declines (Li et al., 2014). Notably, environmental insult exposure during developmental periods, such as prepuberty and adolescence, has a significant impact on neural plasticity and behavior later in life (Encinas et al., 2013; Sanders A. P. et al., 2015). In animals, like rodents and primate, it has been observed that early-life Pb exposure induced cognitive deficit and latent increases in amyloid biomarkers relevant to AD as aged adults (Bihaqi et al., 2014; Liu et al., 2014).

The mammalian SIR2 ortholog, SIRT1, is a ubiquitous and remarkably histone deacetylase. It has shown protective effects in a number of physiological and pathological processes due to its functions in metabolism, stress resistance and genomic stability (Haigis and Guarente, 2006). It is of note that the role of SIRT1 is linked with various proteins which modulating stress response or neuronal apoptosis in Aβ-treated models and age-related diseases (Kumar et al., 2016; Palomera-Ávalos et al., 2017).

The specific SIRT1 activator resveratrol, a naturally occurring phytochemical product in grapes and red wine, has attracted wide attention because of its antioxidant and antiinflammatory effects (Pallauf et al., 2016). Importantly, resveratrol was also shown to mediate neuroprotection *in vitro* and *in vivo* by regulating antioxidant defenses or mitochondrial function (Pallàs et al., 2013; Torres-Pérez et al., 2015). SIRT1 activation by resveratrol may target a myriad of neuronal injury and neurodegeneration paradigms (Herskovits and Guarente, 2014). Of these, both Aβ and Pb are able to potentiate formation of oxidation products that will lead to neurotoxicity and DNA damage (Sanders T. et al., 2015; Navigatore-Fonzo et al., 2017). It is proposed that resveratrol can attenuate Aβ-induced neurotoxicity through activating SIRT1 signaling and autophagy (Deng and Mi, 2016; Wang et al., 2017). Additionally, recent study indicated that resveratrol intervention could revert the reduction of the expression of hippocampal SIRT1 and phosphorylated-cAMP response element-binding protein (pCREB) induced by developmental Pb exposure (Feng et al., 2016). Indeed, these features portray resveratrol as an ideal candidate in response to environmental stress of neurotoxic insult, whether SIRT1 mediate these benefits against chronic Pb exposure is still the subject of continuous experimental support.

Here we studied a possible interplay between Pb exposure during the early life period and the chronic access to resveratrol on spatial learning and memory abilities, and on neurochemical aspects in elder mice. Also we are interested in studying the expression profiles of SIRT1 as signaling molecule in regulation of tissue oxidative status and inducible amyloidogenesis lesion.

## Materials and methods

### Animals and reagents

A total of 48 male C57BL/6 mice (13 ± 2 g, aged 3 weeks) were purchased from the Vital River Laboratory Animal Technology (Beijing, China) and group-housed in accordance with the Guide for the Care and Use of Laboratory Animals published by Ministry of Health of People's Republic of China. All experimental procedures and protocols were reviewed and approved by Life Science Ethics Review Committee of Zhengzhou University.

Lead acetate [Pb(Ac)_2_] with purity of ≥99.5% was purchased from Aladdin Bio-Chem Technology (Shanghai, China). Resveratrol was obtained from Sigma Aldrich (St. Louis, MO, USA). Pb(Ac)_2_ was dissolved into deionized water at a concentration of 0.2%. Resveratrol was diluted in 5% carboxymethyl cellulose sodium (CMC-Na). GSH-Px, MDA and GSH assay kits were purchased from Nanjing Jiancheng Bioengineering Institute (Nanjing, China). ELISA kit for Aβ_1−−40_ was obtained from Biosource International (Camarillo, USA). Antibody against BDNF, TrkB, phosphorylated TrkB (pTrkB, Y515), β-actin and Lamin B was purchased from Abcam Company (Cambrige, UK). Antibodies against 8-hydroxy-2-deoxyguanosine (8-OHdG) and phosphorylated SIRT1(pSIRT1, Ser 47) was obtained from Beijing Biosynthesis Biotechnology (Beijing, China) while SIRT1, AMPK and phosphorylated AMPK (pAMPK, Thr 172) were purchased from Cell Signaling Technology (Danvas, MA, USA).

### Experimental design

**Figure 1:**
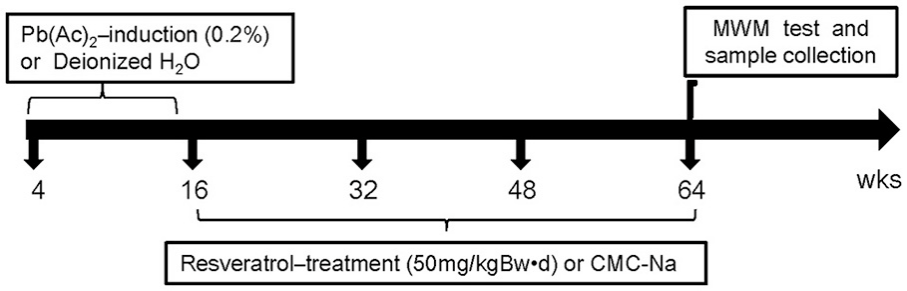
Experimental protocol design. A cohort of C57BL/6 mice were subjected to Pb(Ac)_2_ or resveratrol treatment at various time point.

After 1 week of normal feeding to allow for accommodation, 4-week old mice were divided into four groups of 12 mice each: Control group (Cont), Pb-exposed group (Pb), Pb + Resveratrol group (PbR), Control + Resveratrol group (CR). For Pb group, lead exposure was induced by providing a free drink of 0.2% Pb(Ac)_2_ solution for 12 weeks. For CR group, mice were housed until the age of 16 weeks, and then were gavaged with 50 mg/kgBw•d of resveratrol every other day for another 48 weeks. Both Cont group and Pb group were also administered with the same volume of CMC-Na starting at 16 weeks old. For PbR group, mice were similarly treated with Pb(Ac)_2_ but subject to a 48-week oral gavages' supplementation with resveratrol at the dose of 50 mg/kgBw•d. A timeline with our experimental design can be seen in Figure 1.

### Morris water maze (MWM) test

The mice were subjected to assess spatial learning in the MWM test at the age of 64 weeks, and the behavioral test was performed double blinded according to the standard protocol. Water was made opaque by the addition of 200 g of powdered milk. During the platform trial days, each mouse was trained in the water maze and allowed a maximum of 60 s to find a hidden platform for 5 consecutive days, 4 trials per day with a 30-s interval. On each trial, the mice started from one of the middle of the four quadrants (N, E, S, W) facing the wall of the pool. Escape latencies and swim patterns were digitally monitored. On the 6th day, the probe test was performed, in which the mice were subjected to a 60-s free swim to find the previous location of the platform starting from each of the four quadrants. The pathway that the mice passed through the previous platform quadrant was recorded by a video camera which was connected to a digital-tracking device attached to the computer loaded with the water maze software (Zhenghua Biologic Apparatus Facilities, Huaibei, China).

### Preparation of brain samples

At the end of the behavioral test, the mice were sacrificed with 10% chloral hydrate and brains were removed and rinsed with precooling isotonic saline. Three perfused brain samples from each group were fixed with 4% paraformaldehyde-PBS solution (pH 7.4) at 4°C overnight for further analysis. The remaining hippocampus and cortex were quickly separated and the tissues were stored at −80°C until analysis.

### Biochemical assay

Small portions of cortical tissue were carefully dissected and homogenized with ice-cold buffer (50 mM Tris-HCl buffer, pH7.4) to prepare a 10% homogenate solution. The homogenate was centrifuged (10,000 g, 15 min) and aliquots of supernatant were separated for estimation the activities GSH-Px and catalase (CAT) as well as the levels of MDA and GSH using assay kits. All the operations followed the protocols provided in the kits.

### ELISA kit assay

For brain Aβ_1−−40_, 60 mg of fresh-frozen mouse cortex was serially homogenized into detergent-soluble and guanidine HCl-soluble fractions. All samples were assayed for Aβ_1−−40_ using a commercially available ELISA assay kit according to the manufacturer's instructions.

### Immunohistofluorescence (IHF) analysis for 8-OHdG, SIRT1, and pSIRT1

Paraffin histological sections (5 μm thick) with an average distance of 5 μm apart were obtained from each brain sample. The sections were rinsed and incubated with permeabilization solution, followed by blocking with 10% normal goat serum. IHF staining was performed using primary antibody (rabbit polyclonal antibody 1:400 dilution, at 4°C, overnight) for 8-OHdG or SIRT1, followed by the addition of anti-rabbit Alexa Fluor 488 FITC (Molecular Probes) secondary antibody. The sections were counterstained with 4', 6-Diamidino-2-phenylindole (DAPI, dilution 1:500, Sigma) to identify cellular nuclei. Additionally, mouse monoclonal antibody (dilution 1:200 at 4°C overnight) was used as primary antibody for pSIRT1, followed by the addition of Cy3 goat anti-mouse IgG (Abbkine, CA, USA) at a 1:200 dilution for 2 h at 37°C in the dark. Next, the sections were washed with PBS three times and fluorescence quencher was applied. Subsequently, the images (three images per section) were acquired by using the fluorescent microscope system (Olympus, FV1000, Tokyo, Japan). For negative control experiments, the primary antibodies were omitted. In addition, three sections per brain were used for analyzing the mean fluorescence value of 8-OHdG-labeled cells by Image-Pro Plus software (IPP 6.0) in mice brain from five views under the fluorescence microscope (Nikon, Japan).

### RNA extraction and real-time quantitative PCR

The total RNA from hippocampus and cortex tissues prepared in advance was extracted using TRIZOL reagent (Invitrogen, USA). Then 2 μg of RNA of each sample was transcribed to cDNA using the TaKaRa RNA PCR™ kit (Takara, Japan). The mRNA expression of Bdnf, Sirt1, App, Bace1 and Gadph was inspected using cDNA as a template for amplification utilizing the following primers: Bdnf *IV* (forward primer 5'-CAGAGCAGCTGCCTTGATGTT-3' and reverse primer 5'-GCCTTGTCCGTGGACGTTTA-3'); Sirt1 (forward primer 5'-GAGGTCTCGA TATGTGCTGGA-3' and reverse primer 5'-TTCCTGCAACCTGCTCCAAG-3'); App (forward primer 5'-GACTGACCACTCGACCAGGTTCTG-3' and reverse primer 5'-CTTGTAAGTTGGATTCTCATATCCG-3'); Bace1 (forward primer 5'-CCTACACCCAGGGCAAGT-3' and reverse primer 5'-GGGCAGTAGTAAC TTTGCAGT-3'); and Gapdh (forward primer 5'-CTTCCAGGAGCGAGACC-3' and reverse primer 5'-CGGAGATGATGACCCTTTT-3'). The expression levels of each sample were normalized against Gadph (internal control) and calculated using the comparative CT method (2^−ΔΔCT^).

### Western blotting analysis

Immunochemical analysis of protein level and phosphorylation status was performed by Western blotting in standard conditions. Briefly, equal amounts of samples were separated by 10~12% SDS-PAGE and transferred to PVDF membranes. The membranes were blocked in Tris-buffered saline mixed with Tween-20 (TBST, pH 7.4) containing 5% skim milk for 60 min. Then the membranes were incubated with primary antibodies for desired proteins in blocking buffer at 4°C overnight. β-Actin, β-Tubulin and Lamin B were used as loading control for total, cytosolic and nuclear proteins, respectively. For densitometric analysis, the blots were scanned with Amersham Imager 600 and the pixel intensities of each band of interest were quantified using Image Quant TL (7.0 version, GE Healthcare, USA). Immunoblots shown are representative of three independent biological replicates.

### Statistical analysis

All statistical analyses were performed using the *SPSS* software (version 17.0) and GraphPad Prism 5 for Windows was used for graph fitting. The escape latency data in the MWM test were analyzed by a two-way analysis of variance (ANOVA). Probe trail and other data among multiple groups were determined using one-way ANOVA followed by Tukey's *post-hoc* test. The correlation between the two variables was analyzed by line correlation analysis. All data in the text and figures were expressed as mean ± standard error of the mean (SEM), with *n* representing the number of animals used in each experiment. Statistical significance was defined at the level of *P* < 0.05.

## Results

### Chronic resveratrol treatment ameliorated spatial learning and memory deficits in Pb -exposed mice

**Figure 2:**
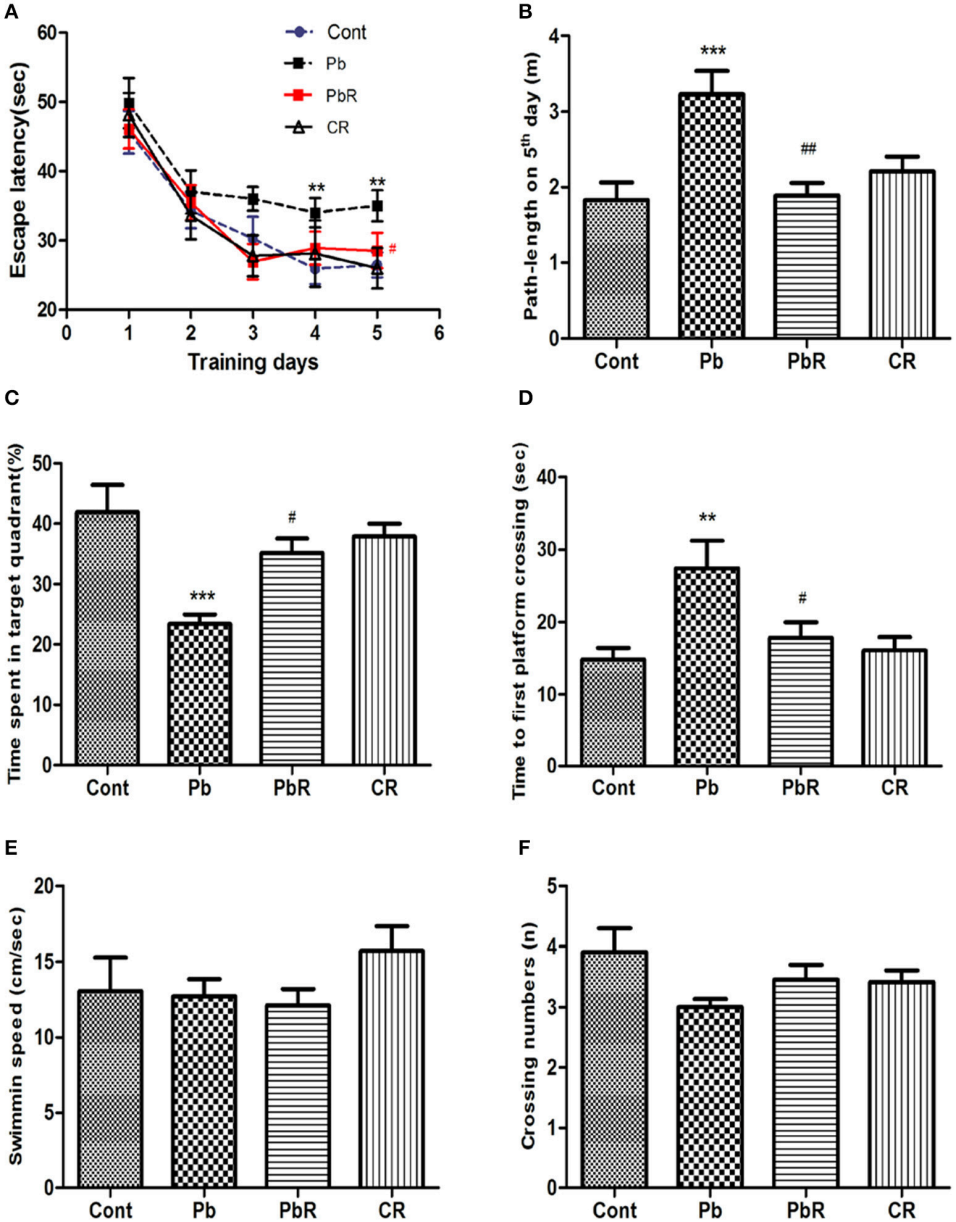
Effects of resveratrol on spatial learning and memory deficits induced by Pb. **(A)** Escape latency of the 5-day acquisition trial for mice of each group; **(B)** Cumulative path-length of acquisition trial on 5th day; **(C)** The mean duration in target quadrant during probe trials; **(D)** The mean time to first platform crossing; **(E)** The mean swimming speed; **(F)** The mean crossing numbers of mice in each group.Values are expressed as mean ± SEM (*n* = 12). ^**^P < 0.01, ^***^P < 0.001 compared to Cont group; ^#^*P* < 0.05, ^##^*P* < 0.01 compared to Pb group.

MWM test was performed to evaluate the effects of resveratrol gavage on impairment of spatial learning and memory in Pb-treated mice and the results were summarized in Figure 2. There was a significant difference in mean latency between training days[*F*_(4, 220)_ = 29.47, *P* < 0.001] and between treatments [*F*_(3, 220)_ = 4.72, *P* < 0.05], but there was no interaction between the factors day and treatment [*F*_(12, 220)_ = 0.38, *P* > 0.05].

The Pb-group mice performed longer latency to reach the hidden platform on day 4 and 5 of acquisition period (*P* < 0.01, Figure 2A) and cumulative path length of 5th day (*P* < 0.001, Figure 2B) compared to control group. Meanwhile, the mice subjected to resveratrol treatment showed a significant decrease in the latency (*P* < 0.05) and the path length (*P* < 0.01) relative to Pb group on day 5.

On day 6 of probe trail, time spent in target quadrant was remarkably reduced in Pb-treated group when compared to the control group (*P* < 0.001, Figure 2C), whereas the time to cross the first non-exit platform was prolonged (*P* < 0.01, Figure 2D). However, these indexes were significantly improved in mice that received resveratrol compared to those that only received Pb (*P* < 0.05 respectively, Figures 2C,D). PbR group had effects similar to those of control group. No significant difference was found in crossing numbers and swimming speed between all groups (*P* > 0.05 respectively, Figures 2E,F).

### Chronic resveratrol treatment improved BDNF expression and TrkB phosphorylation

**Figure 3:**
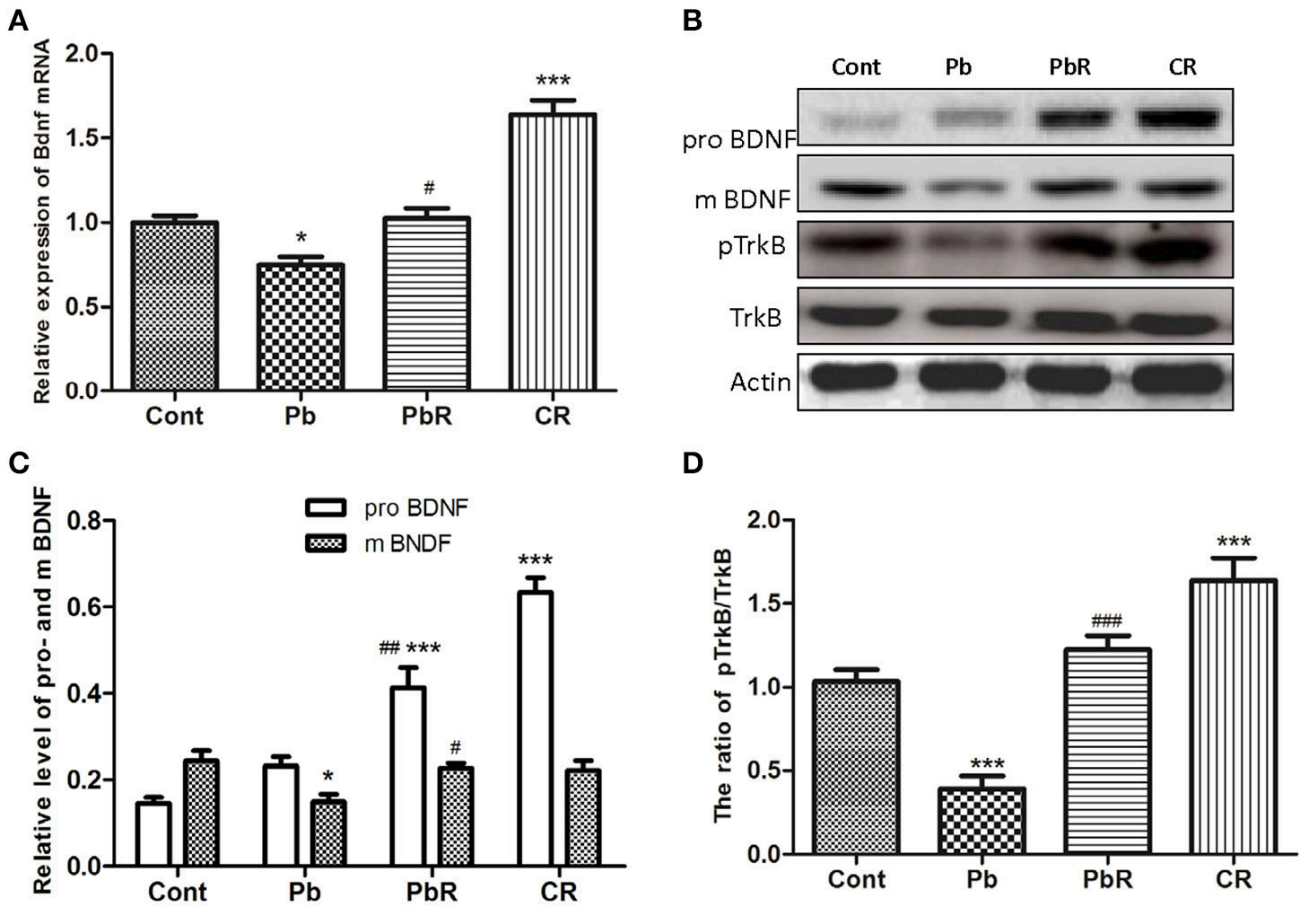
Effects of resveratrol on BDNF expression and TrkB phosphorylation in the hippocampus induced by Pb. **(A)** The endogenous Bdnf transcript levels were determined by RT-qPCR from mice hippocampal homogenates of each group. Quantitative results for each sample normalized by GADPH. **(B–D)** Representative Western blot and quantitative results for pro- and mBDNF and phosphorylated TrkB. The densities are normalized to β-actin. Values are expressed as mean ± SEM (*n* = 6). ^*^P < 0.05, ^***^P < 0.001 compared to Cont group; ^#^*P* < 0.05, ^##^*P* < 0.01, ^###^*P* < 0.001 compared to Pb group.

To confirm our findings from MWM test, we further measured the level of hippocampal BDNF. As shown in Figure 3, RT-qPCR results demonstrated that the Bdnf expression at transcription level had an appreciable reduction after Pb exposure while treatment with resveratrol substantially promoted Bdnf expression (*P* < 0.05 respectively, *n* = 6, Figure 3A). The level of mature BDNF (mBDNF) protein by Western blotting had a similar trend (*P* < 0.05 respectively, Figures 3B,C). In comparison, the level of proBDNF in both PbR and CR group gradually increased compared to control (*P* < 0.001 respectively, Figure 3C), but it did not display a marked increase in the mice of Pb group. The activation of TrkB, known as cognate receptor of BDNF, was also determined by western blotting (Figure 3B). Exposure to Pb resulted in significant declines in pTrkB/TrkB ratio in the hippocampus when compared to control mice (*P* < 0.001, *n* = 6, Figure 3D). Additionally, resveratrol demonstrated the ability to normalize the decline of TrkB activation induced by Pb (*P* < 0.001).

### Resveratrol protects hippocampus from Pb-induced oxidative stress

**Figure 4:**
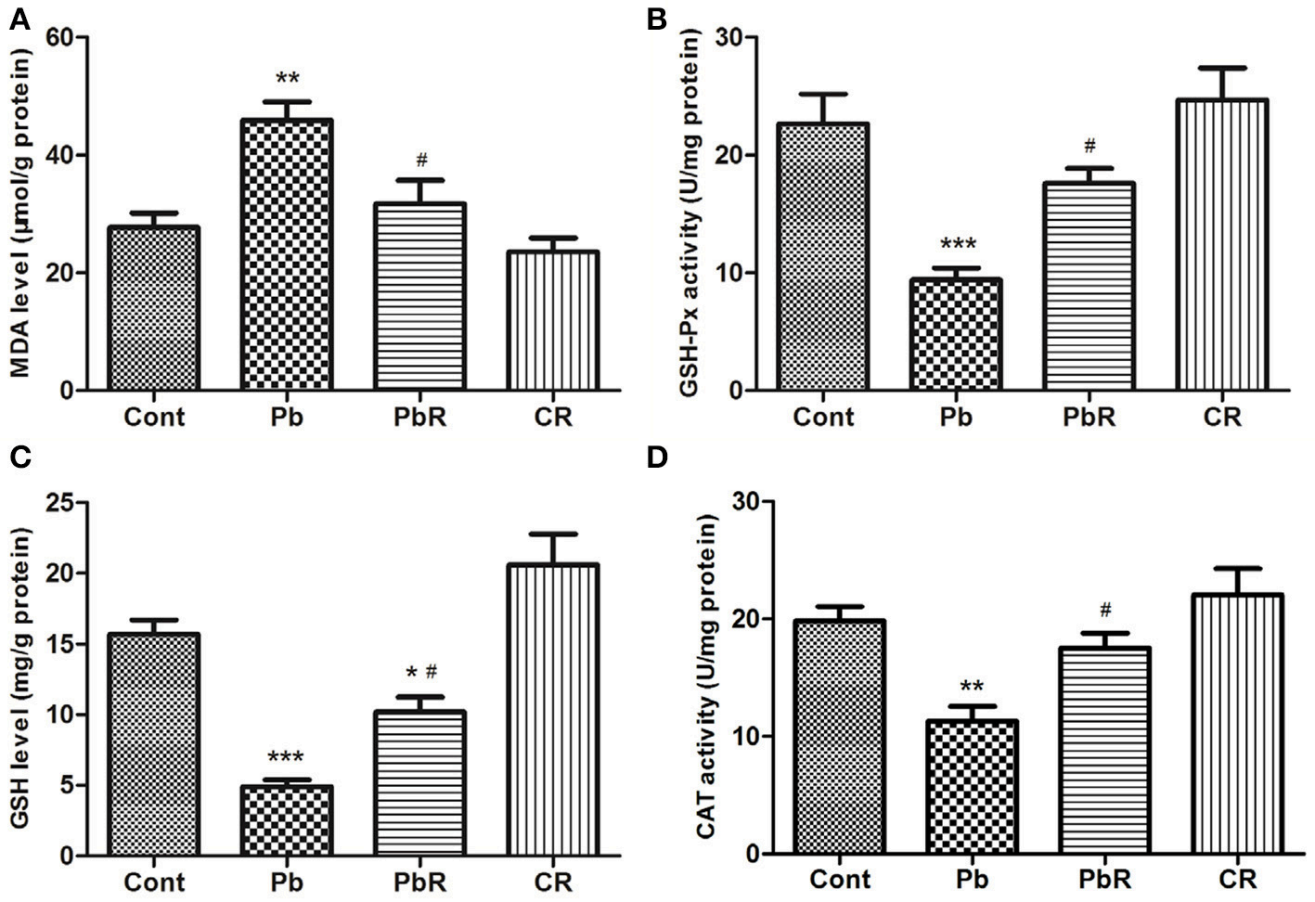
Oxidative stress markers in aged mice. **(A)** MDA level, **(B)** GSH-Px activity, **(C)** GSH level, and **(D)** CAT activity were determined in the cortical tissue of each mice (*n* = 8). ^*^P < 0.05, ^**^P < 0.01, ^***^P < 0.001 compared to Cont group; ^#^*P* < 0.05 compared to Pb group.

**Figure 5:**
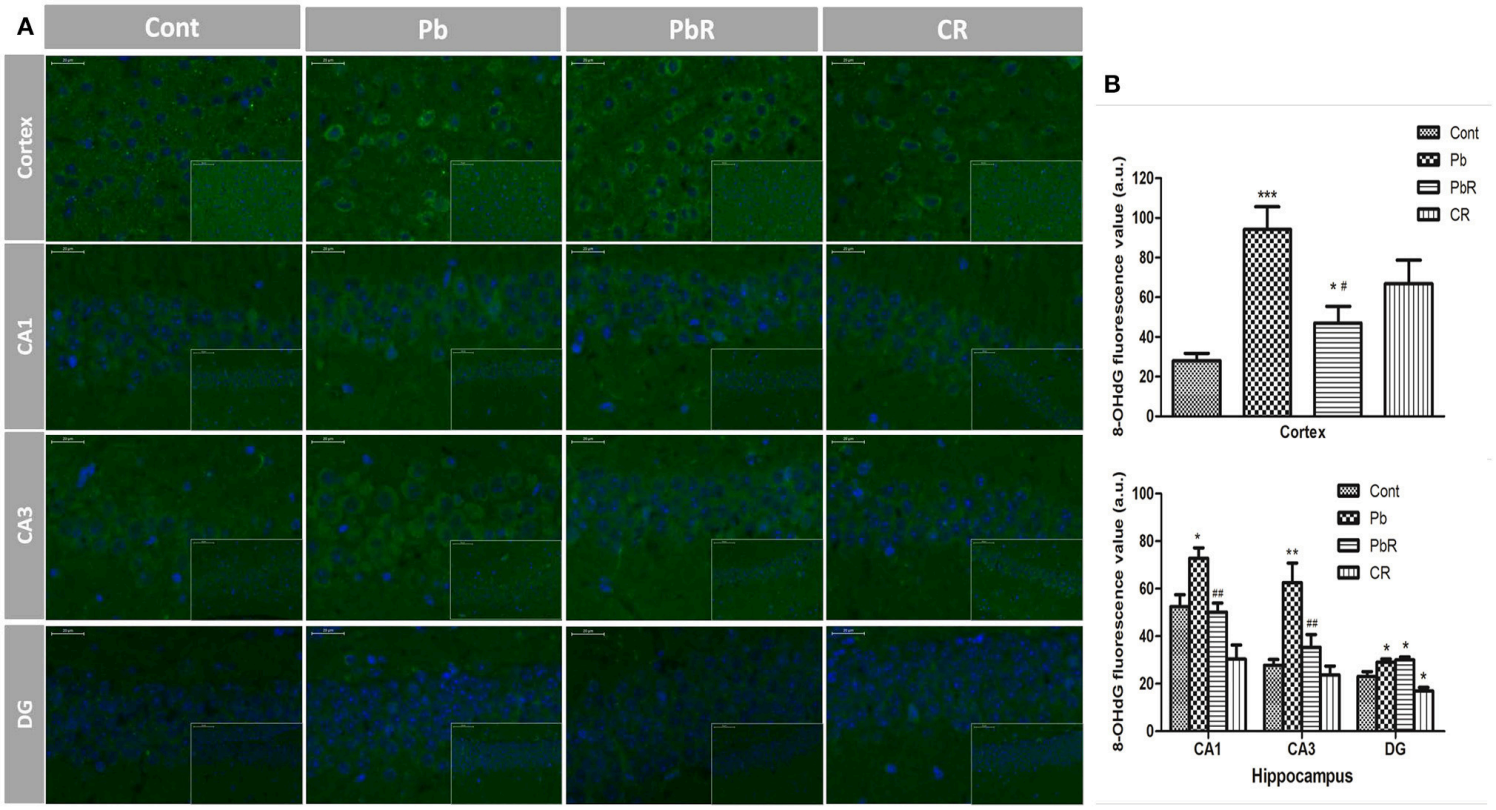
Resveratrol partly restored the Pb-induced oxidative DNA damage. **(A)** Representative photomicrographs of 8-OHdG positive staining by immunofluorescent (IF) method, 8-OHdG is shown in green, DAPI staining in blue; **(B)** Quantitative fluorescence value of 8-OHdG in the cortex and hippocampus of each group (*n* = 3). Values are expressed as mean ± SEM. ^*^P < 0.05, ^**^P < 0.01, ^***^P < 0.001 compared to Cont group; ^#^*P* < 0.05, ^##^*P* < 0.01 compared to Pb group.

Substantial evidences indicate oxidative stress is lightly linked with neurotoxic action of Pb. Hence, we firstly assessed cortical oxidation by measuring pro-oxidant and anti-oxidant biomarker profiles. As shown in Figure 4A, the mice displayed an increase in MDA after Pb exposure (*P* < 0.01, *n* = 8). Meanwhile, there was a significant reduction in cortical activities of GSH-Px and CAT as well as GSH content in Pb group compared to control group (*P* < 0.001 for GSH-Px and GSH, *P* < 0.01 for CAT, Figures 4B–D). In comparison, resveratrol administration led to reversal of high level of MDA and reduction of the above anti-oxidant biomarkers induced by Pb. As a marker of oxidative damage to DNA, 8-OHdG immunoreactivity was also detected and representative images for quantitative analysis of fluorescence value in the sub-region of brain were shown in Figure 5A. After Pb exposure, there were significant increases in the 8-OHdG content in the cortex as well as CA1 and CA3 while fail to alter its content in the DG region (*P* < 0.001 in cortex, *P* < 0.05 in CA1 and DG, *P* < 0.01 in CA3, Figure 5B). Treatment with resveratrol partly depressed the enhancement of 8-OHdG content in the cortex and hippocampus (*P* < 0.05 in cortex, *P* < 0.01 in CA1 and CA3).Taken together, the results indicated that Pb-induced oxidant damage were partly restored following resveratrol treatment.

### Resveratrol elevated SIRT1 expression in nucleus depleted by Pb exposure

**Figure 6:**
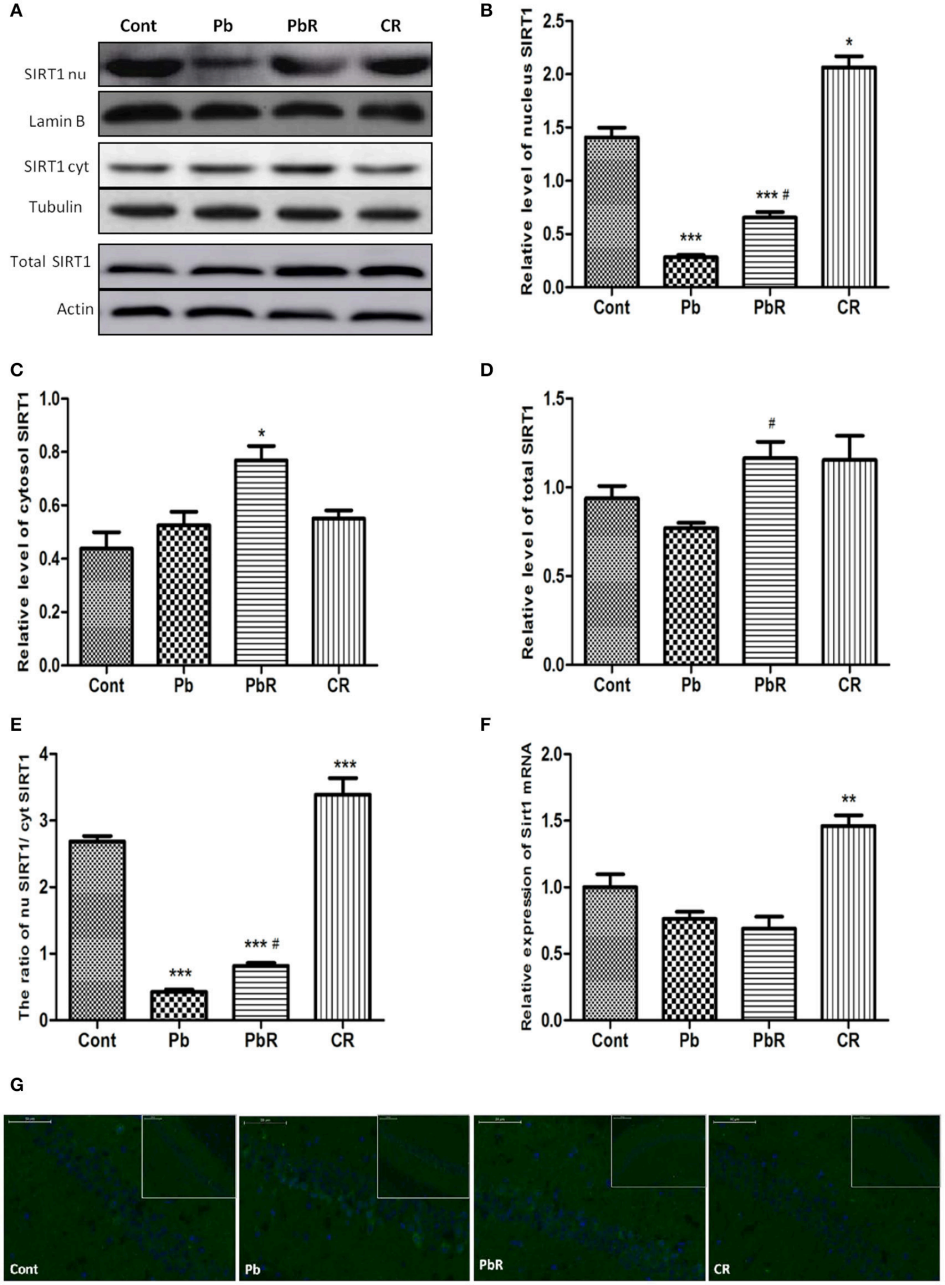
Effects of resveratrol on SIRT1 expression in nucleus depleted by Pb exposure. **(A–E)** Representative Western blot and quantitative results for SIRT1 level in nucleus and cytosol of hippocampus. The densities are normalized to β-actin, β-Tubulin or Lamin B respectively. **(F)** The endogenous Sirt1 transcript levels were determined by RT-qPCR from mice hippocampal homogenates of each group. Quantitative results for each sample normalized by GADPH. Values are expressed as mean ± SEM (*n* = 6). **(G)** Localization of SIRT1 in CA1 region of mice hippocampus observed under fluorescence microscope. SIRT1 is shown in green, DAPI staining in blue. ^*^P < 0.05, ^**^P < 0.01, ^***^P < 0.001 compared to Cont group; ^#^*P* < 0.05 compared to Pb group.

Given that resveratrol was reported to be a potential activator of SIRT1 and could protect mice against Pb-induced oxidative stress, we detected the involvement of SIRT1 in mounting the protective effect of chronic resveratrol using Western blotting and RT-qPCR. As shown in Figure 6, the level of SIRT1 in nucleus was found reduced in Pb group compared to control group (*P* < 0.001, *n* = 6, Figures 6A,B). The rate of SIRT1 level in nucleus/cytosol had a significant difference (*P* < 0.001, Figure 6E) while no alternation of total SIRT1 protein and mRNA level between two groups (Figures 6D,F). In contrast, chronic resveratrol treatment could induce SIRT1 expression in the cell nuclei of PbR group mice (*P* < 0.05) indicating that resveratrol can repress the nucleo-cytoplasmic translocation of SIRT1 induced by Pb exposure (Figures 6A,G).

### Resveratrol promoted SIRT1 phosphorylation in the hippocampus of Pb-exposed mice

**Figure 7:**
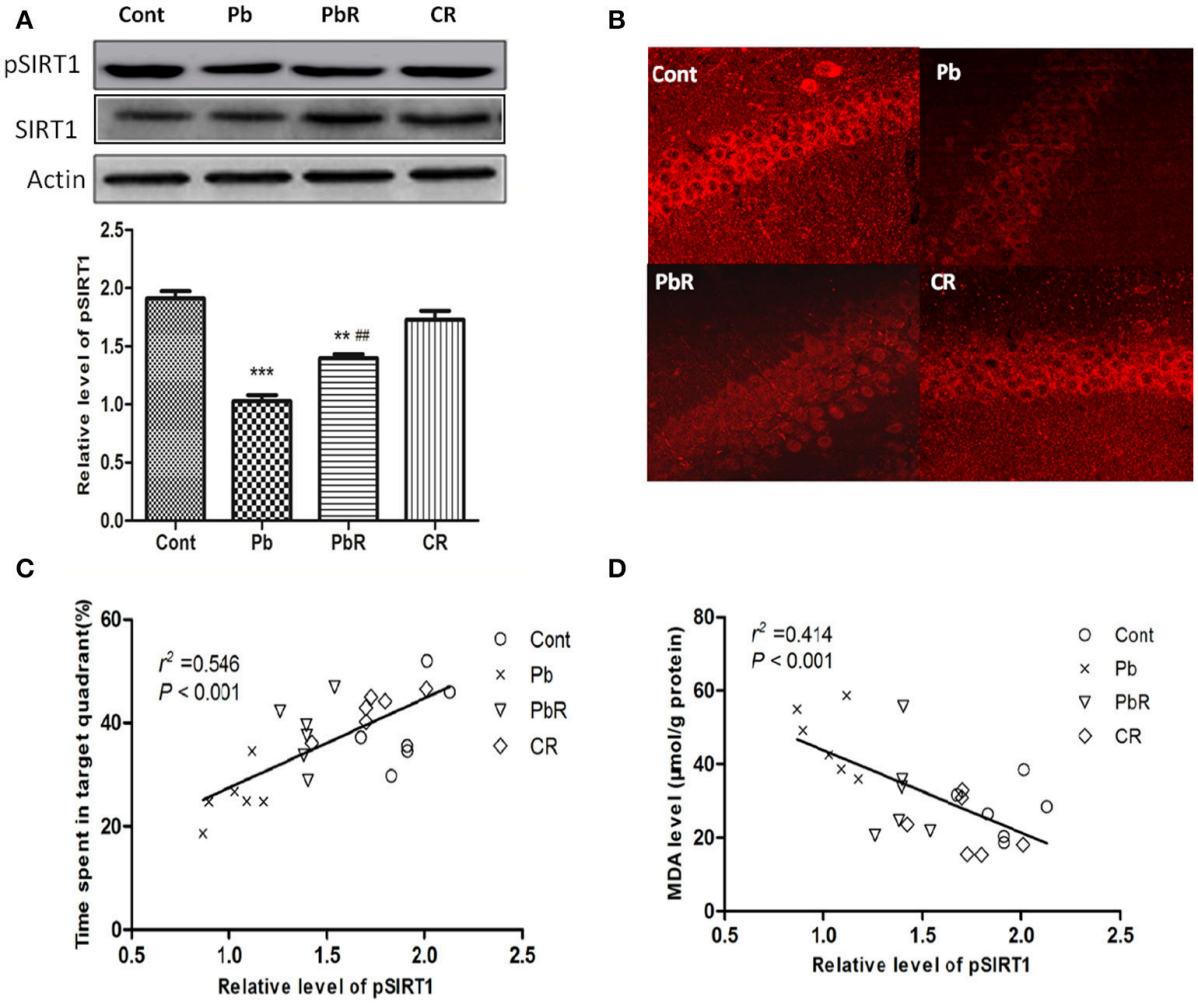
Effects of resveratrol on SIRT1 phosphorylation in the hippocampus of Pb-exposed mice. **(A)** Representative Western blot and quantitative results for pSIRT1 level in the hippocampus. The densities are normalized to β-actin. **(B)** Representative IHF staining with pSIRT1 in CA1 region of hippocampus. **(C)** Correlation analysis of pSIRT1 with probe time in target quadrant in MWM test. **(D)** Correlation analysis of hippocampal pSIRT1 with cortical MDA level (*n* = 6). Values are expressed as mean ± SEM. ^**^P < 0.01, ^***^P < 0.001 compared to Cont group; ^##^*P* < 0.01 compared to Pb group.

Previous studies suggested that phosphorylation of human SIRT1 can affect its subcellular distribution. In view of this, we further investigated whether phosphorylation of SIRT1 was altered during chronic Pb-induced nuclear SIRT1 by Western blotting. As shown in Figure 7, the phosphorylated SIRT1 at Ser47 site was found to be down-regulated in Pb group compared to control or PbR group (*P* < 0.001 or *P* < 0.01, Figure 7A). Additionally, we analyzed the effects of Pb and resveratrol on the phenotypic expression of hippocampal SIRT1 phosphorylation using IHF staining. As shown in Figure 7B, decreased pSIRT1 expression was found in Pb group rather than control group and the presence of resveratrol can induce upregulation of pSIRT1.

We further assessed the relationship between pSIRT1 and spatial learning and memory performance by correlation analysis conducted with GraphPad Prism 5. As shown in Figure 7C, pSIRT1 have positive correlation with probe time in target area. In addition, the hippocampus pSIRT1 expression was significantly and negatively related to cortical MDA content by correlation analysis (Figure 7D).

### Resveratrol counteracted the alterations of phosphorylated AMPK and nuclear PGC-1α induced by Pb

**Figure 8:**
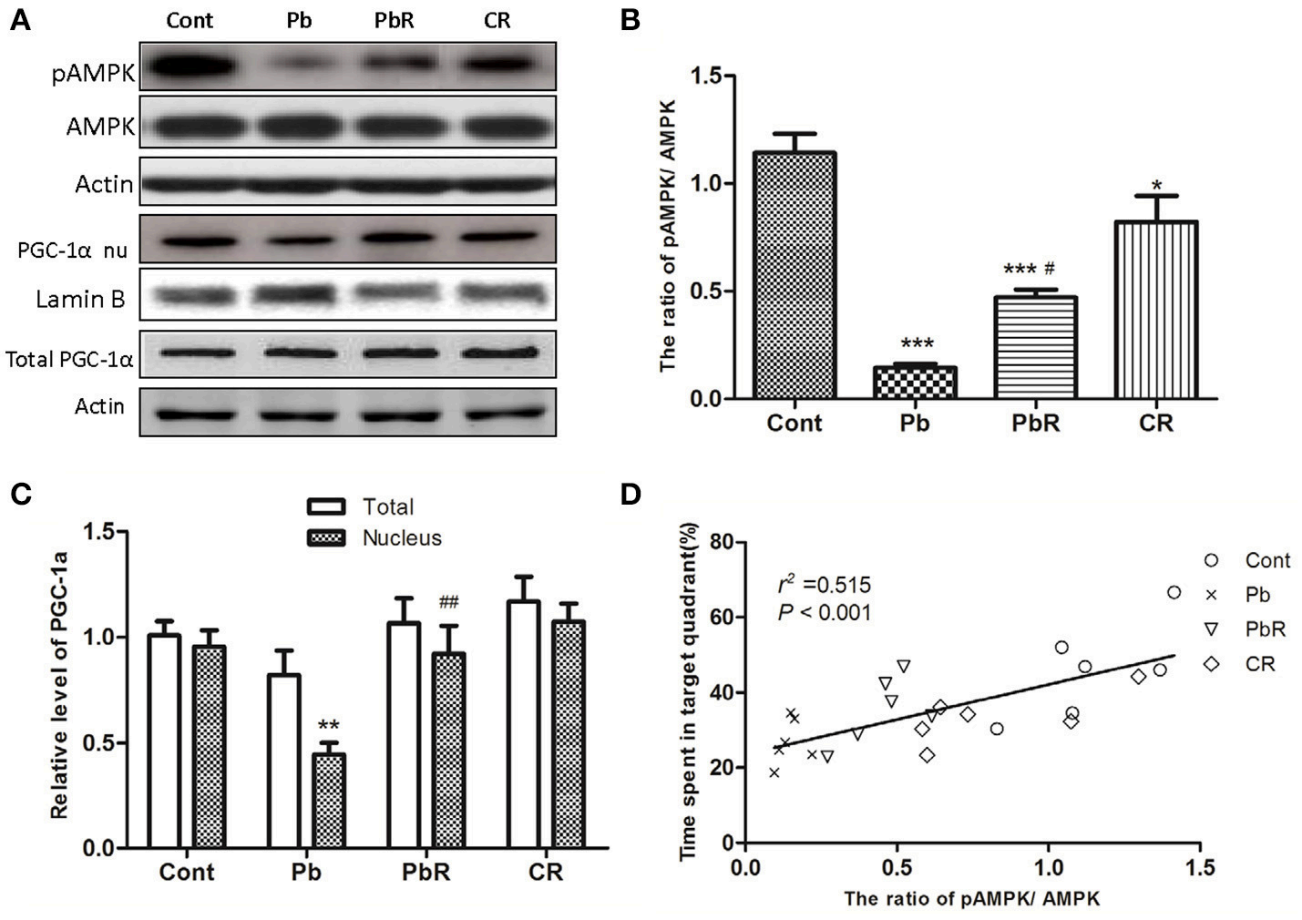
Effects of resveratrol on pAMPK and PGC-1α expression in the hippocampus depleted by Pb exposure. **(A–C)** Representative Western blot and quantitative results for phosphorylated AMPK and nuclear PGC-1α in the hippocampus (*n* = 6). The densities are normalized to β-actin or Lamin B respectively. **(D)** Correlation analysis of pAMPK/AMPK level with probe time in target quadrant in MWM test. Values are expressed as mean ± SEM. ^*^P < 0.05, ^**^P < 0.01, ^***^P < 0.001 compared to Cont group; ^#^*P* < 0.05, ^##^*P* < 0.01 compared to Pb group.

Some evidence has shown that SIRT1 is a crucial regulator of AMPK activation in mitochondrial function, thus we explored whether AMPK was involved in Pb-induced SIRT1 alteration. As shown in Figure 8, compared to control mice, the ratio of pAMPK/AMPK was declined in mice of Pb group(*P* < 0.001, Figures 8A,B). In contrast, resveratrol partly restored the Pb-induced reduction of AMPK phosphorylation in the hippocampus (*P* < 0.05, *n* = 6). Moreover, the expression of nuclear PGC-1α was decreased in mice of Pb group (*P* < 0.01, Figure 8C), whereas no change were determined in total PGC-1α protein. Also, a recuperative increase of nuclear PGC-1α in PbR group was observed (*P* < 0.01, *n* = 6). In addition, the level of pAMPK/ AMPK had positive correlation with probe time in target area by correlation analysis (Figure 8D).

### Resveratrol repressed the amyloidogenic processing induced by Pb

**Figure 9:**
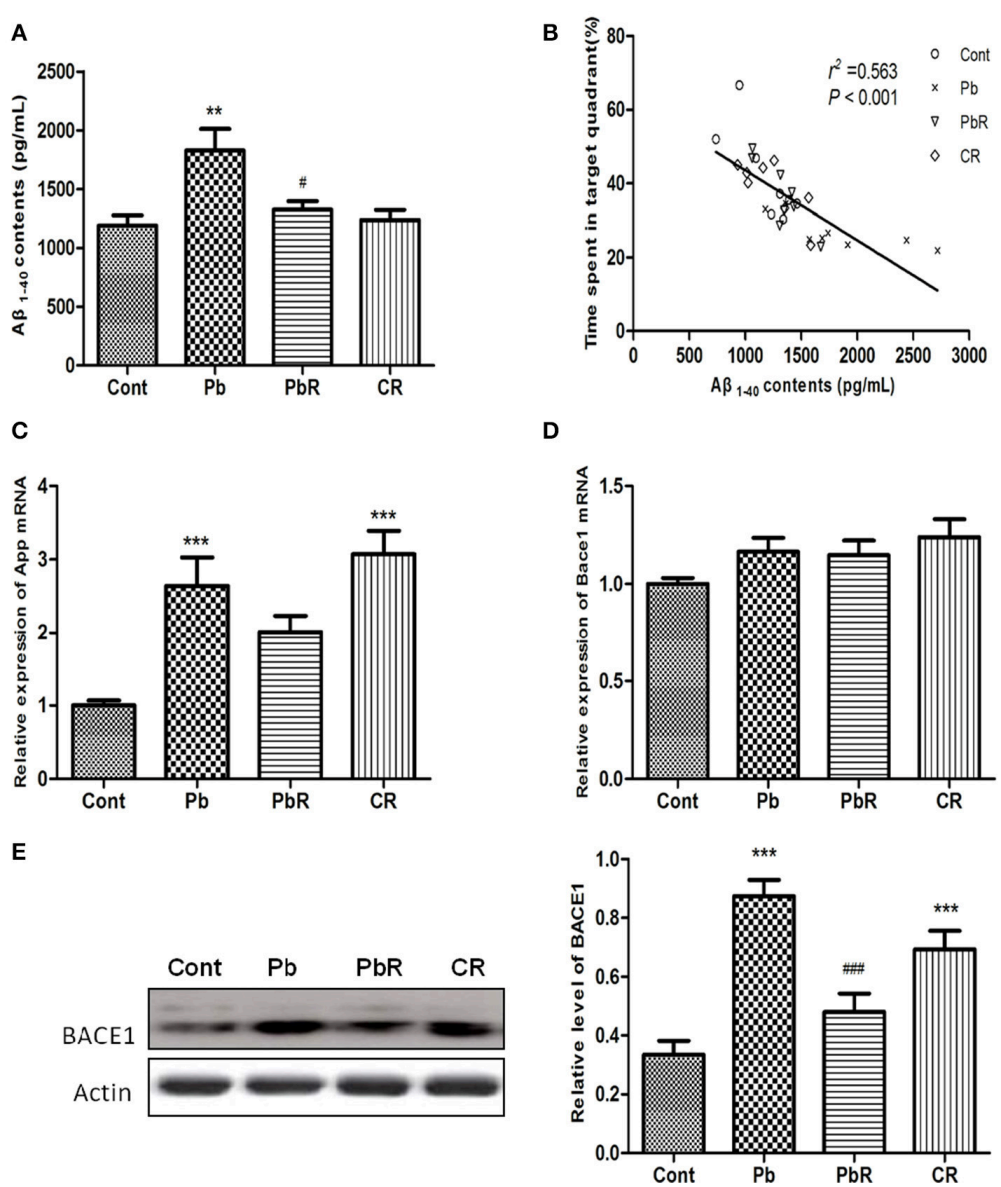
Effects of resveratrol on amyloidogenic processing in the cortex of Pb-exposed mice. **(A,B)** Aβ_1−40_ content and its correlation with probe time in target quadrant in MWM test. **(C,D)** The endogenous App and Bace1 transcript levels were determined by RT-qPCR from mice cortical homogenates of each group. **(E)** Representative Western blot and quantitative results for BACE1 level. The densities are normalized to β-actin. Values are expressed as mean ± SEM (*n* = 8). ^**^P < 0.01, ^***^P < 0.001 compared to Cont group, ^#^*P* < 0.05, ^###^*P* < 0.001 compared to Pb group.

Aβ is known to play a critical pathogen role in AD and is involved in Pb-induced cognitive impairment; we therefore measured Aβ_1−−40_ in the cortex by ELISA. As shown in Figure 9A, the Aβ_1−−40_ level of mice from Pb group was significantly higher than that of control group (*P* < 0.01, *n* = 8). In contrast, resveratrol could suppress Aβ_1−−40_ overproduction when compared to the Pb group(*P* < 0.05). Furthermore, there was a negative correlation of cortical Aβ_1−−40_ content with probe time in target area of Pb-exposed mice(*P* < 0.01, Figure 9B). As shown in Figures 9C–E, chronic Pb exposure increased the mRNA of App (*P* < 0.01, *n* = 8) and protein level of BACE1 (*P* < 0.001, *n* = 8) whereas no change was determined in mRNA of Bace1. In contrast, resveratrol demonstrated the ability to normalize the increase of BACE1 level following Pb exposure (*P* < 0.001, Figure 9E). The data suggested that resveratrol could partly abolish abnormal production of Aβ_1−−40_ and the amyloidogenic processing induced by Pb.

## Discussion

Chronic exposure to Pb mostly by inhalation and ingestion raises its accumulation in the CNS, especially in the basal choroid plexus and hippocampus, promoting degeneration of neurons and an interference with synapse formation (Baranowska-Bosiacka et al., 2012; Wang et al., 2013). Epidemiologic and experimental studies demonstrated that Pb exposure increased the risk for both neurodevelopmental disorders (Kasten-Jolly et al., 2012) and neurodegenerative disease (Weisskopf et al., 2007; Basha and Reddy, 2010; Athanasopoulos et al., 2016). We previously showed that rodent with developmental Pb exposure was at increased risk for cognitive dysfunction, mainly mediated by enhanced neuroinflammation (Li et al., 2009, 2014; Liu et al., 2014). Similarly, the current data also demonstrated that spatial learning and memory was impaired in chronic Pb-exposed elder mice which displayed longer escape latencies and less time in the target quadrant in the Morris water maze. Since BDNF-TrkB signaling was critically important in maintaining hippocampal-dependent cognition (Gupta et al., 2013); we subsequently found that Pb reduced the levels of molecular systems related to the neurotrophic factor mBDNF, including phosphorylation of the TrkB receptor. Furthermore, Pb-exposed mice also displayed deficient antioxidant activities and extensive DNA damage, with resultant cognitive decline.

As a multi target polyphenol, resveratrol modulate intracellular effectors associated with oxidative stress, neuroinflammation, synaptic plasticity as well as amyloidogenesis (Mercè et al., 2013). Considerable evidences support a protective role of resveratrol on preventing cognitive deficits in normal aging and neurodegenerative disorders. Here we found that resveratrol treatment was able to attenuate the negative effect of Pb on spatial learning and memory. Accordingly, the mBDNF levels as well as activation of TrkB were rescued by resveratrol. Recent study also illustrated that resveratrol effectively suppressed the decline of hippocampal pCREB, a downstream target of BDNF, after developmental Pb exposure (Feng et al., 2016). In addition, resveratrol also exhibits potent antioxidant capacity due to its ability to remove free radicals and metals and to activate endogenous antioxidant enzymes (Liu et al., 2015; Whitehouse et al., 2016). In view of this, we examined how resveratrol modulate a number of the pro-oxidant and anti-oxidant biomarker (MDA, GSH, GSH-Px, CAT, and 8-OHdG) on Pb-induced oxidative stress process in the present study. The effect of resveratrol was demonstrated by the restoration of above oxidative stress markers, and the restoration was paralleled by upregulation of BDNF-TrkB pathway in the hippocampus. Take all together, the present results indicated that systemic administration of resveratrol was able to protect and even rescue Pb-induced spatial learning and memory impairment, an effect that could be explained, at least in part, by its purported antioxidant.

Sirtuins (SIRTs) is structurally important and its distinct role in the regulation of neuronal survival is also disclosed. Many studies, in recent years highlight SIRT1 is thought to function as nutrient sensors, which mediate a neuroprotective action in various models of toxicity. Several investigators pointed out experimental traumatic brain injury (TBI) or subarachnoid hemorrhage induced oxidative stress and SIRT1 alteration in injured rats (Zhang et al., 2016; Yang et al., 2017). This specific inhibition of SIRT1 expression may promote the activation of post-TBI-induced mitochondrial apoptosis pathway (Yang et al., 2017). Conversely, overexpression of SIRT1 reduced axonal loss in hippocampus and inflammation effects in the spinal cord (Nimmagadda et al., 2013). Moreover, SIRT1 has been implicated in modulating general cognitive enhancement and synaptic plasticity through the regulation of BDNF during normal brain aging as well as onset of neurologic disorders (Gao et al., 2010; Chen et al., 2016). In view of this, role of SIRT1 was assessed in Pb-induced toxicity in the hippocampus of mice. Interestingly, chronic Pb exposure reduced the mRNA of SIRT1 without affecting its total protein levels in the aging brain, in contrast to findings from earlier study that reported hippocampal SIRT1 expression in transcript and protein levels to be downregulated during Pb exposure (Feng et al., 2016). The discrepancy could be due to different intervention process of resveratrol relative to Pb treatment. And, more importantly, the transcript alteration in SIRT1 leads us to distinguish its protein expression between cytoplasmic and nuclear forms (Tong et al., 2013). In this study, nuclear SIRT1 level of Pb-exposed mice was downregulated in the hippocampus, and SIRT1 phosphorylation after chronic Pb exposure was also blunted, indicating that, in addition to reduced nuclear SIRT1 expression, the hippocampus could not mount as a robust SIRT1 response to toxic Pb. In turn, resveratrol treatment was effective to sequester SIRT1 into the nucleus after early-life Pb exposure which resulted in an increase in pSIRT1. Additionally, our results further showed probe time in target area of the MWM test was extended in proportion to increase in pSIRT1. As a whole, these findings revealed that nuclear localization and phosphorylation of hippocampal SIRT1 were important for the action of resveratrol on maintaining spatial learning and memory performance in cases of neurotoxic Pb exposure.

As described above, resveratrol was able to attenuate Pb-induce oxidative stress by promoting antioxidant defenses. The present result also showed that MDA level in the cortex was inversely proportional to pSIRT1 levels, thus emphasizing the SIRT1-mediated antioxidant role of resveratrol. It is noteworthy that recently, resveratrol is also described as a direct activator of AMPK involving in regulating antioxidant response coordinated with the expression of key mitochondrial regulators, such as PGC-1α and SIRT1 (Higashida et al., 2013; Zhang et al., 2014). Here, results of these three regulators in cellular components of hippocampus exhibited significant changes between control and Pb-exposed mice. Of these, the fact that phosphorylation levels of SIRT1 and AMPK in Pb-exposed mice were restored by resveratrol treatment reflected the action of resveratrol on SIRT1/AMPK activation. Moreover, it is reported that resveratrol can promote SIRT1/AMPK activation (Valenti et al., 2016) and is implicated in neural plasticity (Torres-Pérez et al., 2015; Dias et al., 2016; Tian et al., 2016). Similar to our results for pSIRT1, the present study showed probe time in target area of the MWM test was also positively correlated with the ratio of pAMPK/AMPK which enhanced the link between cognition and resveratrol involving AMPK. Moreover, SIRT1 can enhance PGC-1α activation, which acts as a regulator in resistance to oxidative stress and mitochondrial integrity (Cantó and Auwerx, 2009; Hofer et al., 2014). Treatment with resveratrol in the present study also effectively revert nuclear PGC-1α and thus is associated with SIRT1/AMPK activation exhibiting SIRT1-mediated modulation of oxidative stress. Together these data, so far, provide an indication for the importance of SIRT1 signaling pathway in modulating restorative role of resveratrol against neurotoxic effect of Pb.

However, the hypothesis that resveratrol acts particularly via this specific SIRT1/AMPK signaling pathway should be interpreted with caution. In the present study, resveratrol treatment alone did not produce any change in pSIRT1 level or on anti-oxidative effects but improved anti-oxidative effects and spatial learning and memory impairment induced by Pb, suggesting that resveratrol might act via other mechanisms not yet elucidate here. In addition, the interaction between SIRT1 and AMPK deserves to be further explored.

Increased intracellular Pb has been linked to an increase in secreted levels of the AD-causing Aβ and a number of epidemiological studies have shown its potential as a risk factor in cognitive decline of the elderly (Weisskopf et al., 2007; Peters et al., 2010). In the present study, exposure to Pb increased the content of Aβ as well as amyloid precursor protein (APP) transcript level. Furthermore, increased levels of Aβ in the cortex of Pb-exposed mice may be associated with poor learning and memory performance as observed, suggesting a causative relation between the increased Aβ_1−−40_ and the Pb-induced cognitive impairment. Additionally, BACE1 which cleaves APP at its β-site is rate limiting for the neuronal APP processing (Kuruva and Reddy, 2017). Elevated protein levels of BACE1 in elder mice with prior to Pb exposure seem to enhance the action of Pb on beta cleavage of amyloidogenic pathway in accordance with previous report (Bihaqi et al., 2014). In our opinions, apart from the antioxidant and cognitive improvement role of resveratrol, we also demonstrated the ability of resveratrol to modify the production of Aβ, affording neural resilience in response to lead. Some studies pointed out that SIRT1 may influence both Aβ and neurofibrillary tau pathology in the transgenic mouse models of AD (Herskovits and Guarente, 2014). As a robust activator of SIRT1, resveratrol has shown to possess anti-amyloidogenic activity and to modulate intracellular effectors such as AMPK (Vingtdeux et al., 2010). As showed in the present study, the treatment with resveratrol could result in an apparent decline in Aβ content as well as BACE1 protein level in Pb-exposed mice. Our observation supports the action of resveratrol on activation of anti-amyloidogenic pathway. Based on these results, it appears that resveratrol is essential for engaging signals related to amyloidogenesis in chronic Pb-exposed mice. In addition, emerging evidence suggested that SIRT1 could directly activate ADAM10 which mediates cleavage of soluble APPα (Donmez et al., 2010; Wang et al., 2016). Thus, the link between amyloidogenesis and resveratrol involving SIRT1 also need to be further researched in depth.

In conclusion, our study demonstrated that the capacity of the SIRT1 agonist resveratrol to counteract the effects of early-life Pb exposure on spatial learning and memory of elder mice. More importantly, we first found SIRT1 nucleocytoplasmic translocation and reduced pSIRT1 in hippocampus of chronic Pb-treated mice. According to our results, prolonged administration of resveratrol elicited significant improvement of spatial learning and memory which was associated with activating SIRT1 via modulation of oxidative stress. In addition, resveratrol was effective to attenuate the effects of Pb on neurochemical markers associated with hippocampal synaptic plasticity (mBDNF and TrkB) and cortical APP processing (Aβ_1−−40_ levels and BACE1). These findings emphasize the potential of SIRT1 activator as an efficacious dietary intervention to downgrade the Pb-induced neurotoxic lesion.

## Ethics statement

This study was carried out in accordance with the recommendations of international and national related biomedical research ethics. The protocol was approved to perform by the Life Science Ethics Review Committee of Zhengzhou University.

## Author contributions

LZ: Study Design, data interpretation, manuscript preparation, literature search. RT: Data collection, data interpretation, literature search, manuscript preparation. YW: Statistical analysis, data Interpretation, manuscript preparation. YH: Data collection, literature search. XL: Data collection, literature search. XC: Data collection, literature search. YY: Data collection. WL: literature search, funds collection. HH: Study design, data interpretation, manuscript preparation, funds collection.

### Conflict of interest statement

The authors declare that the research was conducted in the absence of any commercial or financial relationships that could be construed as a potential conflict of interest.
